# Continuing Professional Development–Medical Imaging

**DOI:** 10.1002/jmrs.870

**Published:** 2025-02-26

Maximise your continuing professional development (CPD) by reading the following selected article and answer the five questions. Please remember to self‐claim your CPD and retain your supporting evidence. Answers will be available via the QR code and published in JMRS—Volume 72, Issue 4, December 2025.

### Occupational Burnout in Nuclear Medicine Technologists Working in Australia and New Zealand—Results of a Multi‐National Survey

M. Shields, D. James, L. McCormack, *Journal of Medical Radiation Sciences* (2025), https://doi.org/10.1002/jmrs.834.

1. What are the three dimensions that define occupational burnout?
  - Emotional exhaustion, depersonalisation, reduced personal accomplishment
  - Depression, emotional exhaustion, lack of empathy
  - Emotional exhaustion, physical exhaustion, depression
  - Lack of empathy, anxiety, absenteeism
2. In this study, which Australian state had the highest mean occupational burnout score for nuclear medicine technologists?
  - New South Wales
  - Victoria
  - Queensland
  - Western Australia
3. Which Australian state had the highest number of registered nuclear medicine technologists, according to the Medical Radiation Practice Board of Australia (MRPBA) at the time this study was conducted?
  - New South Wales
  - Victoria
  - Queensland
  - Western Australia
4. Is susceptibility to occupational burnout influenced by an individual's personality and personal characteristics?
  - No
  - Yes
5. According to the study authors, what is a possible reason for the Australian state having the highest mean occupational burnout score?
  - High staff turnover rates in healthcare facilities
  - Lack of professional development opportunities for nuclear medicine technologists
  - There is no undergraduate nuclear medicine degree offered in this state
  - Limited access to mental health resources for healthcare workers

## Answers

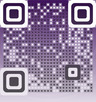

Scan this QR code to find the answers.
